# Informed Consent for Endoscopic Biliary Drainage: Time for a New Paradigm

**DOI:** 10.3390/medicina58030331

**Published:** 2022-02-22

**Authors:** Marco Spadaccini, Cecilia Binda, Alessandro Fugazza, Alessandro Repici, Ilaria Tarantino, Carlo Fabbri, Luigi Cugia, Andrea Anderloni, on behalf of the Interventional Endoscopy & Ultra Sound (I-EUS) Group

**Affiliations:** 1 Digestive Endoscopy Unit, Department of Gastroenterology, Humanitas Clinical and Research Center, IRCCS, 20089 Rozzano, Milan, Italy; alessandro.fugazza@humanitas.it (A.F.); alessandro.repici@hunimed.eu (A.R.); andrea.anderloni@humanitas.it (A.A.); 2 Department of Biomedical Sciences, Humanitas University, 20090 Pieve Emanuele, Milan, Italy; 3 Digestive Endoscopy and Gastroenterology Unit, Department of Gastroenterology, AUSL Romagna, Morgagni-Pierantoni Hospital and Bufalini Hospital, 47100 Forli, Forlì-Cesena, Italy; cecilia.binda@gmail.com (C.B.); carlo.fabbri@auslromagna.it (C.F.); 4 Digestive Endoscopy and Gastroenterology Unit, Department of Gastroenterology, Istituto Mediterraneo per i Trapianti e Terapie ad Alta Specializzazione (IsMeTT/UPMC), 90127 Palermo, Italy; itarantino74@gmail.com; 5 Gastroenterology and Digestive Endoscopy Department, Azienda Ospedaliero Universitaria Sassari, 07100 Sassari, Italy; luigi.cugia@aousassari.it

**Keywords:** biliary tract, pancreatobiliary, intervention EUS

## Abstract

Endoscopic retrograde cholangiopancreatography (ERCP) is considered as the first option in the management of malignant biliary obstruction. In case of ERCP failure, percutaneous transhepatic biliary drainage (PTBD) has been conventionally considered as the preferred rescue strategy. However, the use of endoscopic ultrasound (EUS) for biliary drainage (EUS-BD) has proved similarly high rates of technical success, when compared to PTBD. As a matter of fact, biliary drainage is maybe the most evident paradigm of the increasing interconnection between ERCP and EUS, and obtaining an adequate informed consent (IC) is an emerging issue. The aim of this commentary is to discuss the reciprocal roles of ERCP and EUS for malignant biliary obstruction, in order to provide a guide to help in developing an appropriate informed consent reflecting the new biliopancreatic paradigm.

## 1. Introduction

Endoscopic retrograde cholangiopancreatography (ERCP) is considered as the first option in the management of malignant biliary obstruction, with rates of successful deep cannulation ranging from 89% to 92% using conventional techniques [1,2,3]. Common causes of ERCP failure may include periampullary diverticulum or ampullary distortion due to malignant infiltration [4], nevertheless advanced endoscopic techniques (i.e., double wire-guided technique, pre-cut, transpancreatic papillary septotomy) have shown to improve cannulation rates by up to 97% in such cases [5,6]. Sometimes the papilla remains non-accessible at all because of gastric outlet obstruction (GOO) or surgically altered gastrointestinal (GI) anatomy due to different (benign or malignant) conditions [4,7]. In case of ERCP failure, percutaneous transhepatic biliary drainage (PTBD) has been conventionally considered as the preferred rescue strategy because of its high success rate. However, the significant rate of adverse events (i.e., tube dislodgement/occlusion, cholangitis) significantly contribute to reducing the quality of life of our patients [8,9].

In this regard, endoscopic ultrasound (EUS) for biliary drainage (EUS-BD) was first performed in 2001 by Giovannini et al. [10] and, since then, it has shown rates of technical success comparable to PTBD. Further, lower risks of both adverse events and need for reintervention were reported in several studies and a meta-analysis [11,12,13,14,15]. As a matter of fact, biliary drainage is maybe the most evident paradigm of the increasing interconnection between ERCP and EUS [16]. The complementarity between the two techniques nowadays is becoming more and more evident, changing the essence of biliopancreatic endoscopy itself, with implications for different aspects beyond the endoscopic room. Obtaining an adequate informed consent (IC) is for sure one of those aspects. This troublesome, but underestimated issue, is often taken for granted, even if remaining a fundamental legal and ethical principle before any (endoscopic) procedures.

The aim of this commentary is to discuss the reciprocal roles of ERCP and EUS for malignant biliary obstruction, in order to provide a guide to help in developing an appropriate informed consent reflecting the new biliopancreatic paradigm.

## 2. From General Principles to Our Starting Point

The principle of IC is based on the human right for autonomy and self-determination [17,18]. However, it is not only required by ethical aspects, but also incorporated in legal requirements. A number of legal judgements have been raised from problems in achieving fully-informed consent, and these judgements have clarified the interpretation of consent with particular emphasis on the provision of information [19]. Appropriate IC procedures must include information about the (1) *mechanisms of action*, (2) the balance between *benefits and risks*, and (3) the *alternative treatments*. Moreover, the presentation must be as *clear* as possible [20,21] in order to result first in the patient understanding what the procedure will involve, and then in his/her agreeing (or declining).

Focusing on the management of malignant biliary obstruction (MBO), in order to match these ideal requirements for an optimal IC process, we first had to face several questions [22] to set a steady starting point. At the present, several national and international endoscopy societies shared IC form templates to be signed before different endoscopic procedures (upper endoscopy, colonoscopy, EUS, ERCP, …) in order to propose a standardized approach to our patients. Even if considering the perspective of a common behavior as the winning strategy for such issue, all the proposed forms show a “technique-based” design. If it is adequate for most pure diagnostic procedures, the advances in interventional endoscopy are rapidly increasing the number of weapons available to the endoscopist to achieve one aim (i.e., to resect a GI lesion, to perform a biliary drainage), thus, as happened in surgery in the last years, we should probably change the way of thinking endoscopy and related IC, moving to a “goal-based” IC, overcoming the concept of “technique-based” ones [19].

For instance, EUS-BD is already considered a treatment option according to the ESGE Guidelines [23,24], as evidence supports an integrated ERCP/EUS approach. Is it still acceptable to re-scheduled a second procedure if the standard ERCP approach fails due to the lack of an explicit IC? As a matter of fact, we do not wake the patient up, ask for the consent and reschedule a patient before using an advanced endoscopic technique (i.e., fistulotomy) if the standard transpapillary drainage fails. We just include that strategy within the ERCP borders, and do it when needed. Why should we not widen those borders? Who fixed them? After all, if we look deep into it, choledocoduodenostomy could be considered as an EUS-guided pre-cut differing from what we are used to do with a needle knife by a couple of centimeters and, possibly, by the type of the stent.

This approach would permit one to provide the best chance to reach our goal in a single endoscopic session, avoiding a second sedation, longer hospital stays, and inconvenient costs [25,26,27,28].

With the aim to create a common document addressing this troublesome issue, through a modified Delphi process [29], an ad-hoc commission of the Interventional Endoscopy & Ultra Sound group (I-EUS) created a dedicated IC form focused on the aim of the procedure, namely biliary drainage, more than on technical aspects [22]. Different mechanisms of action, namely ERCP and EUS-guided procedures, with their main risks, were included in an easy-to-understand, illustrated form, in order to obtain a more conscious understanding.

## 3. Mechanisms of Action

### 3.1. Endoscopic Retrograde Cholangiopancreatography (ERCP)

At the moment, ERCP is considered as the first option in the management of malignant biliary obstruction. It consists in using a duodenoscope to reach the second portion of the duodenum. The bile duct is then accessed through the papilla and the drainage is obtained by placing a biliary stent to restore the bile flow toward the duodenum [23]. As mentioned above several advanced techniques have been developed, over the past years, to improve cannulation success rates, including precut (papillotomy vs. fistulotomy), the double-guidewire technique, and pancreatic duct access-assisted cannulation [5,30].

### 3.2. EUS-Guided Rendezvous

When the papilla is accessible, EUS-guided rendezvous (EUS-RV) may be considered as a salvage technique in case deep cannulation cannot be achieved [31]. This approach can be performed using either an extrahepatic or transhepatic access using a linear echoendoscope puncturing the dilated biliary system with a 19-gauge needle, obtaining a cholangiogram, and advancing guidewire downstream through the papilla into the duodenum. Thus, the distal end of the guidewire might be grasped and withdrawn via the accessory channel of the scope and a conventional ERCP is performed. Otherwise, biliary cannulation may be re-attempted using a standard duodenoscope along the guidewire [32].

### 3.3. EUS-Guided Choledochoduodenostomy

EUS-guided choledochoduodenostomy (EUS-CDS) results in the connection of the duodenum and the dilated common bile duct (CBD) [33]. After failed ERCP, it may be used in patients with distal biliary obstruction. The CBD is identified by a linear echoendoscope and accessed through a 19-gauge needle. A biliary guidewire is then coiled into the biliary tree and the newly created tract is dilated over the guidewire (balloon dilator vs. cystotome) before proceeding to stent placement. If an electrocautery-enhanced (EC) system is being used, access, tract dilation and stent placement are all performed simultaneously. Following initial experiences with plastic stents, the high rates of complications (42.86% vs. 13.08% [34]) suggested the use of metallic ones. Further technical improvements were achieved by providing anchorage across non-adherent luminal structures through using a fully covered lumen-apposing self-expanding metal stent (LAMS). Moreover, as already mentioned, the use of an EC-LAMS may help in reducing stent deployment time [35,36,37].

### 3.4. EUS-Guided Hepaticogastrostomy

EUS-guided hepaticogastrostomy (EUS-HGS) is a feasible option when transpapillary or transduodenal forms of biliary drainage are prevented because of GOO and surgically altered GI anatomy. With a linear echoendoscope, a dilated left intrahepatic bile duct is identified from the stomach, and then punctured. Thus, a cholangiogram is performed in order to confirm needle placement, then a guidewire is advanced downstream into the CBD, followed by stent placement through the gastric wall.

### 3.5. EUS-Guided Gallbladder Drainage

EUS-GBD can be performed to manage a patient with distal MBO as a rescue treatment when neither ERCP nor other EUS-BD techniques are feasible. In order to permit an effective procedure, the biliary obstruction is distal to the cystic duct takeoff, and the cystic duct patency should be confirmed [38,39,40]. Once the gallbladder is identified by a linear echoendoscope from either duodenal bulb or gastric antrum, it is accessed through a 19-gauge needle (followed by guidewire coiling, tract dilation and stent placement) or directly using electrocautery-enhanced (EC) systems, decreasing the procedure time as mentioned for EUS-CDS.

**Figure 1:**
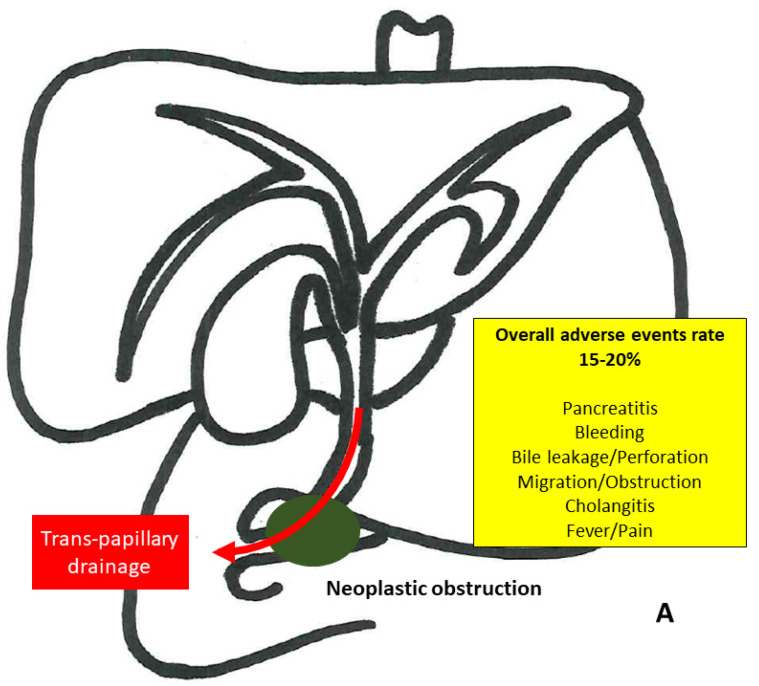

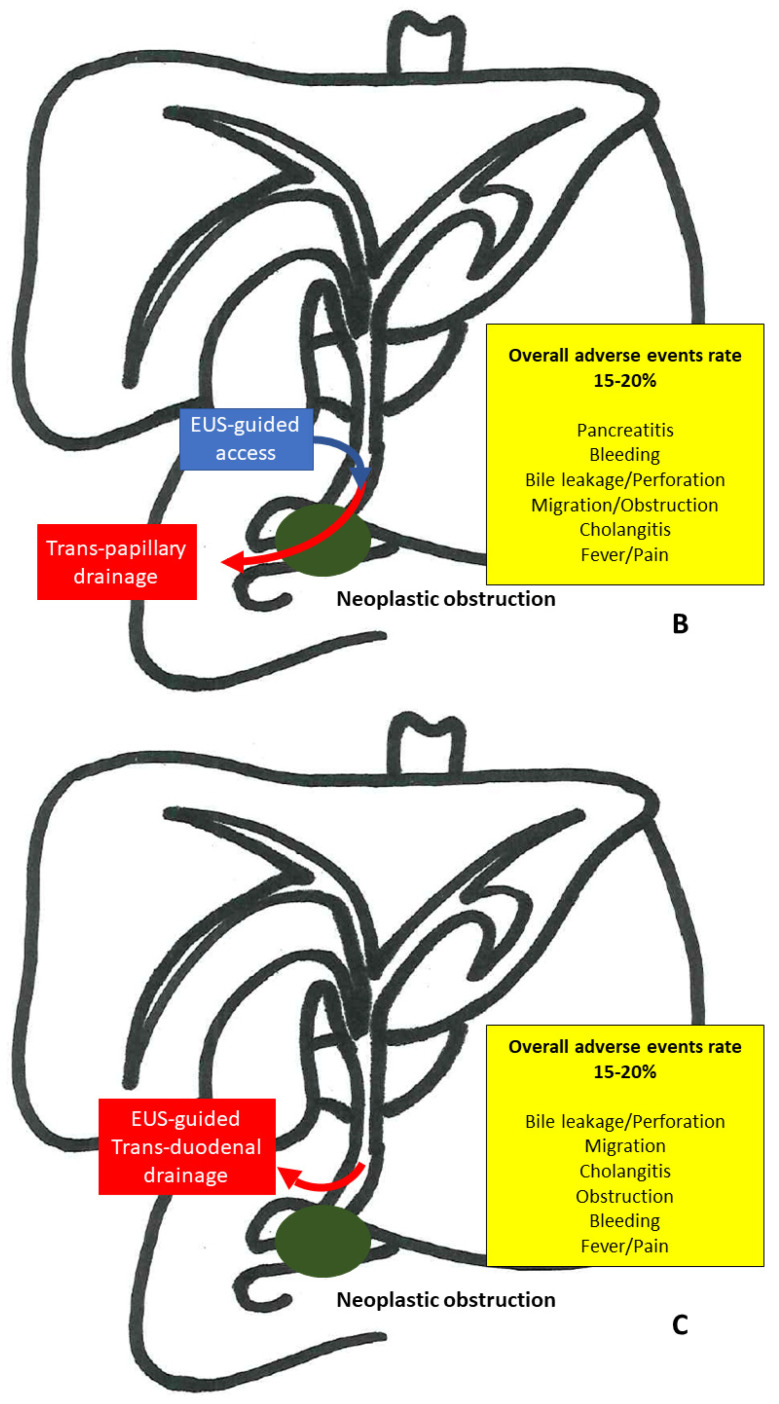

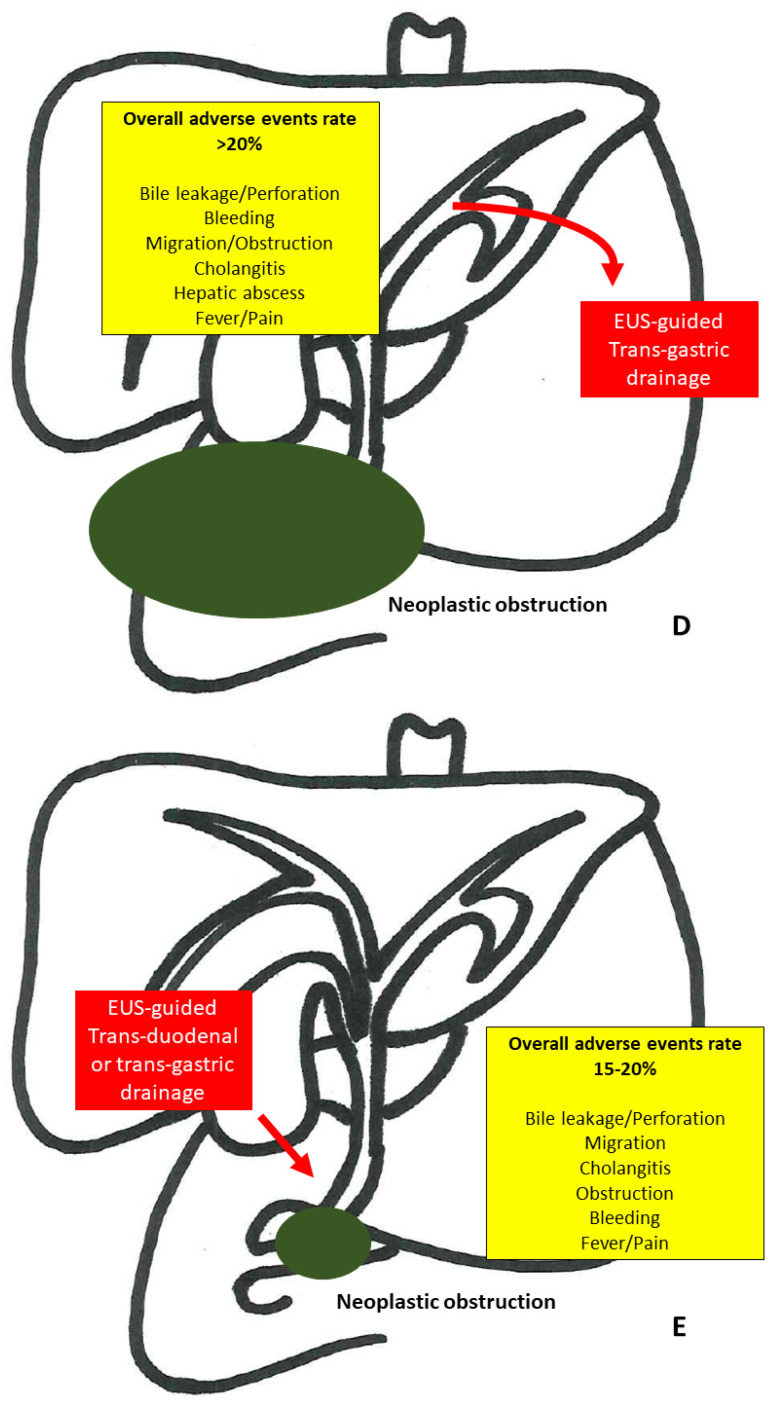
(**A**) Endoscopic Retrograde Cholangiopancreatography (ERCP); (**B**) EUS-Guided Rendezvous; (**C**) EUS-Guided Choledochoduodenostomy; (**D**) EUS-Guided Hepaticogastrostomy; (**E**) EUS-Guided Gallbladder Drainage.

As already mentioned, from the patient point of view, the complexity and the similarities among the procedures require proper illustrations for a more conscious understanding (Figure 1). Further, this figure was made to clearly report the risk of adverse events (AEs) of the different procedures. As a matter of fact, if AEs have been widely investigated for ERCP, available literature lacks conclusive definitions and classifications of AEs for EUS-guided techniques. For instance, there is still heterogeneity in defining what is “early” and “delayed” in case of adverse events such as bleeding and/or stent migration [41], and only a few studies are able to grade severity of AEs according to a shared score such as ASGE Lexicon [42]. Recent comprehensive reviews [43,44] reported adverse event rates between 10% to 20% for EUS-based treatment approaches, which included bleeding (4.03%), bile leakage (4.03%), pneumoperitoneum (3.02%), stent migration (2.68%), cholangitis (2.43%), abdominal pain (1.51%), and peritonitis (1.26%) [43]. However, simplified data based on the most recent evidences were reported in Figure 1, in order to provide a useful tool, as evidence-based as possible, for counseling support.

**Figure 2:**
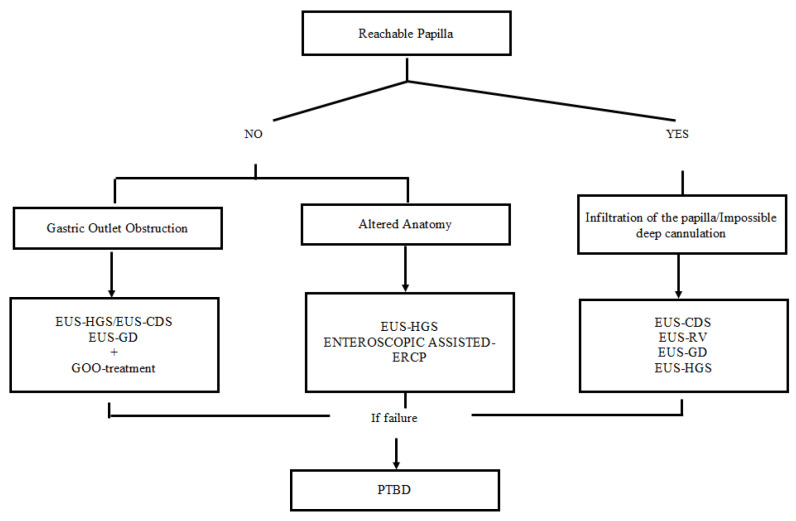
The proposed behavior in case of ERCP failure. EUS-GD may represent a possible rescue strategy in case of the failure of conventional EUS-guided approaches when cystic duct patency has been confirmed. Endoscopic Retrograde Cholangiopancreatography (ERCP); EUS-Guided Rendezvous (EUS-RV); EUS-Guided Choledochoduodenostomy (EUS-CDS); EUS-Guided Hepaticogastrostomy (EUS-HGS); EUS-Guided Gallbladder Drainage (EUS-GD), percutaneous transhepatic biliary drainage (PTBD).

If different mechanisms of action and possible risks must be stated explicitly, considering the evolving scenario in interventional EUS, it may still not be the time for reporting in the IC form a unique step-by-step approach because of the risk of being rapidly overcome by more and more recent evidence. Considering the purpose of this commentary, we reported in Figure 2 the behavior we proposed in case of ERCP failure, aware that local expertise have a strong role in choosing the preferred strategy.

The best EUS-guided technique still needs to be chosen on a patient-based approach, and several studies have already been questioning the role of ERCP as first line treatment in case of MBO, showing similar technical and clinical success when compared to EUS-BD [45,46,47,48,49]. In the future, considering that the risk of procedure-related pancreatitis is theoretically absent in EUS-BD, it may be used as the first line palliative modality in selected cases where the expertise is available.

## 4. Conclusions and Future Perspective

In the medical field, problems with consent may occur because clinicians sometimes undervalue the need of patients for information, and they may feel pressured into consenting to a procedure or too scared to do it, because of an inadequate dialogue. This can be mainly caused by over-emphasis of either the benefits or the risks of a particular treatment, and shortage of time. This easy to use tool for improving our consent form for biliary drainage may facilitate, but not replace, the role (and the time) of the clinicians who need to consider personally discussing the planned procedure with patients.

Looking at the future, the development of a goal-driven informed consent for biliary drainage may be the forerunner of a different way of thinking for the consent forms in endoscopy, and endoscopy itself, since intricate situations may require manifold approaches. For instance, advanced endoscopic resections (ESD vs. EMR vs. FTR vs. …), the management of gastric outlet obstruction (dilation vs. stent placement vs. EUS guided gastro-enteric anastomosis) and emergency procedures may only be the most obvious matches for this paradigm. In this regard, in order not to relegate these issues as a matter of bureaucracy, we do not want to chase the reality after it already happened, but take advantage of these insights to delineate the path we are aiming toward. Thus, if, as in this case, our aim is biliary drainage, we can imagine this consent form as the first step toward a multidisciplinary form instructing the patient that, in the context of a hybrid suite, he/she will have the problem solved with the best possible approach (ERCP/EUS/PTBD) by an endoscopist, or an interventional radiologist (or, why not, a hybrid clinicians) in case of ERCP/EUS failure. The steps to be done are many, but the path does not seem so steep anymore.

## Data Availability

Not applicable.

## Abbreviations

Endoscopic retrograde cholangiopancreatography (ERCP), gastric outlet obstruction (GOO), gastrointestinal (GI), percutaneous transhepatic biliary drainage (PTBD), endoscopic ultrasound (EUS), endoscopic ultrasound for biliary drainage (EUS-BD), informed consent (IC), malignant biliary obstruction (MBO), Interventional Endoscopy & Ultra Sound group (I-EUS), EUS-guided rendezvous (EUS-RV), EUS-guided choledochoduodenostomy (EUS-CDS), electrocautery-enhanced (EC), lumen-apposing self-expanding metal stent (LAMS), EUS-guided hepaticogastrostomy (EUS-HGS).
